# Adenoviruses Associated with Acute Respiratory Diseases Reported in Beijing from 2011 to 2013

**DOI:** 10.1371/journal.pone.0121375

**Published:** 2015-03-27

**Authors:** Meng Chen, Zhen Zhu, Fang Huang, Donglei Liu, Tiegang Zhang, Deng Ying, Jiang Wu, Wenbo Xu

**Affiliations:** 1 Beijing Centres for Disease Control and Prevention, No. 16, Hepingli Middle Street, Dongcheng District, Beijing, 100013, People’s Republic of China; 2 WHO WPRO Regional Reference Measles/Rubella Laboratory, National Institute for Viral Disease Control and Prevention, Chinese Centre for Disease Control and Prevention, No.155, Changbai Road, Changping District, Beijing, 102206, People’s Republic of China; University of Hong Kong, HONG KONG

## Abstract

**Background:**

Adenovirus is one of the most common causes of viral acute respiratory infections. To identify the types of human adenoviruses (HAdVs) causing respiratory illness in Beijing, a sentinel surveillance project on the viral aetiology of acute respiratory infection was initiated in 2011.

**Principal findings:**

Through the surveillance project, 4617 cases of respiratory infections were identified during 2011-2013. Throat swabs (pharynx and tonsil secretions) were collected from all the patients, and 15 different respiratory viruses were screened by multiplex one-step PCR method. 45 were identified as adenovirus-positive from sporadic and outbreak cases of respiratory infection by a multiplex one-step RT-PCR method, and a total of 21 adenovirus isolates were obtained. Five HAdV types among three species, including HAdV-3 (species HAdV-B), HAdV-4 (species HAdV-E), HAdV-7 (species HAdV-B), HAdV-55 (species HAdV-B), and an undefined HAdV type (species HAdV-C) were identified. The comparison results of the penton base, hexon, and fiber gene sequences of the Beijing HAdV-3, HAdV-4, HAdV-7, and HAdV-55 strains in this study and those from the GenBank database indicated significant spatial and temporal conservation and stability of sequences within the genome; however, the phylogenetic relationship indicated that both strain BJ04 and strain BJ09 isolated in 2012 and 2013, respectively, may have recombined between HAdV-1 genome and HAdV-2 genome within species HAdV-C, indicating intraspecies recombination.

**Conclusions:**

This study confirmed that at least 5 HAdV types including HAdV-3, HAdV-4, HAdV-7, HAdV-55 and an undefined HAdV type were co-circulating and were the causative agents of respiratory tract infections in recent years in Beijing. HAdV-3, HAdV-4, HAdV-7, and HAdV-55 showed the apparent stability of the genomes, while intraspecies recombination was identified in strain BJ04 and BJ09. The recombinants carrying penton base gene of HAdV-1 as well as hexon and fiber genes of HAdV-2 might be a novel type of HAdV worthy of further study.

## Introduction

Human adenoviruses (HAdVs) belong to the genus *Mastadenovirus* within the family *Adenoviridae* [1]. Adenoviruses are non-enveloped, icosahedral, double-stranded DNA viruses with genomes of 26–45 kb [1]. The viral capsid is composed of two types of capsomeres: the hexon and the penton (which consists of the penton base and the fiber). Antigens at the surface of the virion are mainly type-specific [2,3]. Hexons are involved in neutralization, and fibers in neutralization and haemagglutination-inhibition. A recombinant that has a unique combination of these three regions (penton base; hexon loops; fiber knob) derived from previously recognized genotypes will be assign a new genotype (http://hadvwg.gmu.edu).

Traditionally, the only basis for recognizing a new type of HAdV is by serology, and on the basis of their biological properties, HAdVs have been classified into 7 species (Human *mastadenovirus* A to G, HAdV-A to HAdV-G), including 52 human HAdV types, which are formally recognized by the International Committee on Taxonomy of Viruses (ICTV) [4,5]. In addition, novel HAdV genotypes (HAdV-53 to HAdV-68) were recently identified based on their bioinformatics and genomic analysis of the complete viral genome sequences (http://hadvwg.gmu.edu). Novel HAdV strains may arise from mutations or recombination among the different types of HAdVs [6].

HAdV can cause a variety of clinical diseases such as acute respiratory disease [7], gastroenteritis [8], and keratoconjunctivitis [9], which vary depending on the cell tropism of the viruses. Among the HAdV-associated respiratory diseases, viruses in species HAdV-B (HAdV-3, 7, 11, 14, 16, 21, 50, 55), species HAdV-C (HAdV-1, 2, 5, 6), and species HAdV-E (HAdV-4) [10–14] are recognized as the main pathogens responsible for the respiratory tract infection.

As the capital city of China, Beijing covers an area of 16,800 km^2^ with a large population of more than 19.72 million (Chinese Statistics Bureau, 2011). In order to elucidate the spectrum of the viral aetiology of acute respiratory infections and provide basic data to guide local disease prevention and control measures, a sentinel surveillance project on the viral aetiology of acute respiratory infections was initiated and sponsored by the Beijing Municipal Health Bureau in 2011. Adenovirus is one of the most common causes of viral acute respiratory infections. In this study, our primary aim was to identify the types of HAdV causing respiratory illness in Beijing since 2011, to avoid the overuse of antibiotics and to improve the level of diagnosis and treatment of respiratory viral disease especially HAdV associated disease in hospitals, and to provide scientific basis for prevention and control of HAdV causing respiratory illness.

## Material and Methods

### Specimen collection and identification

This study did not involve human experimentation; the only human material used in this study was throat swab specimens collected from cases with respiratory tract infection during the implementation of the surveillance project on viral aetiology of acute respiratory infection. This study was approved by the second session of the Ethics Review Committee of the National Institute for Viral Disease Control and Prevention in China CDC. Written informed consent for the use of the clinical samples has been obtained from all patients involved in this study.

Pharynx and tonsil secretions of the patients were wiped with disinfection long cotton swabs with gently action, and after samples collection, all samples were transported under a cold chain and preserved at −80°C for further identification. A multiplex one-step reverse transcription-polymerase chain reaction (PCR) was performed to screen for 15 different respiratory viruses (respiratory syncytial virus A and B, influenza virus A and B, parainfluenza virus 1–4, human adenovirus, human enterovirus, human rhinovirus, human metapneumovirus, human bocavirus, and human coronavirus NL63-229E and OC43-HKU1) simultaneously by using a commercial kit (Seeplex RV 15 ACE Detection kit; Seegene, Inc., Seoul, Korea) [15]. Adenovirus-positive specimens were cultured and further analysed.

### Cell culture and virus isolation

Virus isolation for the adenovirus-positive specimens was performed by using HEp-2 cell lines (from American Type Culture Collection, ATCC Number CCL-23) following the standard protocol [13]. Cells inoculated with clinical samples were incubated at 37°C for 7 days. If no cytopathic effect was observed, the culture was used to inoculate fresh cells for up to 2 additional passages; the cultures with adenovirus-like cytopathic effects were passaged again to confirm the presence of the virus.

### Determination of the nucleotide sequences of penton base, hexon, and fiber gene

The viral DNA was extracted from infected cells by using a QIAamp DNA mini kit (Qiagen, Valencia, CA, USA) according to the manufacturer’s instructions. For the typing of HAdV, the penton base, hexon, and fiber gene sequences were obtained for all HAdV strains; PCR was performed with the Platinum PCR SuperMix (Invitrogen) following the manufacturer instructions. The primer pairs designed to amplify and sequence the penton base, hexon, and fiber gene sequences are listed in Table 1. The amplification products were analysed with capillary gel electrophoresis by using the QIAxcel DNA High Resolution Kit (Qiagen, the Netherlands). After the PCR products were purified with a QIA Gel Extraction Kit (Qiagen, Valencia, CA, USA), the amplicons were bi-directionally sequenced using the Sanger sequencing method (BigDye Terminator, Version 3.1, Cycle Sequencing kit; Life Technologies, Grand Island, NY, USA) and an ABI PRISM 3130 genetic analyser (Applied Biosystems, Foster City, CA, USA).

**Table 1:** Primers used for amplification and sequencing of the entire penton base, hexon, and fiber gene.

Primer	Sequences (5′-3′ orientation)	Position^*a*^
HAdV-2-Penton-1F	GTGTGGGAGGACGATGACTC	13,961–13,980
HAdV-2-Penton-1R	GTTGGTCGGAGCGCTTCTT	15,948–15,966
HAdV3-Penton-1F	GGGCGCATGTTGTAAAAGTAA	13,803–13,823
HAdV3-Penton-1R	GGTGCTGGGTAGAGCGTATG	15,630–15,649
HAdV4-Penton-1F	ATGTGGGACGATGAGGATTC	13,567–13,586
HAdV4-Penton-1R	GTCAATGACGGCGTCCAC	15,608–15,625
HAdV7-Penton-1F	GATGATAGCAGCGTGTTGGA	13,699–13,718
HAdV7-Penton-1R	GGTGCTGGGTAGAGCGTATG	15,595–15,614
HAdV55-Penton-1F	GATGATAGCAGCGTGTTGGA	13,515–13,534
HAdV55-Penton-1R	CACGGGATGTTGGGTAGAAC	15,442–15,461
HAdV-2-Hexon -1F	AAGCACACTGAACAGCATCG	18,690–18,709
HAdV-2-Hexon -1R	CACCACTCGCTTGTTCATGT	20,393–20,412
HAdV-2-Hexon -2F	AGATGAAACTTTTGCAACACGT	20,211–20,232
HAdV-2-Hexon -2R	CGTATTGACTATGGCGCAGG	21,893–21,912
HAdV3-Hexon-1F	AGTACTCTGAACAGCATCGT	18,258–18,277
HAdV3-Hexon -1R	TAGGTGGCGTGTACTTGTAA	19,860–19,879
HAdV3-Hexon-2F	ACCGATGACGCTAATGGATG	19,695–19,714
HAdV3-Hexon -2R	TATGGCGCAGGCGAGCTTGT	21,415–21,434
HAdV4-Hexon -1F	TGACGCACACGGACGAACC	17,773–17,791
HAdV4-Hexon -1R	AGAGGGCAACATTGGCATAG	19,545–19,564
HAdV4-Hexon -2F	ATGGTGTGGAGGATGAATTG	19,334–19,353
HAdV4-Hexon -2R	ACCGGCCGTATTGACGAT	21,138–21,155
HAdV7-Hexon -1F	CTGAACAGCATCGTGGGTCT	18,219–18,238
HAdV7-Hexon -1R	ACTCGCCCGTTCATGTACTC	19,837–19,856
HAdV7-Hexon -2F	CGTCGAGGATGAACTGCCTA	19,560–19,579
HAdV7-Hexon -2R	CAGTGTTGACTATGGCGCAG	21,363–21,382
HAdV55-Hexon -1F	GTGCAAAGTGTAAAACGCCG	18,085–18,104
HAdV55-Hexon -1R	GGTGGTGGTTGAATGGGTTG	19,810–19,829
HAdV55-Hexon -2F	ATACACCCCGTCCAATGTCA	19,672–19,691
HAdV55-Hexon -2R	CGCTTATCGTAGGTTCCCAA	21,194–21,213
HAdV-2-Fiber-1F	CCTTTCCTTCCTCCCAACTC	30,902–30,921
HAdV-2-Fiber-1R	TGTGGTGGTGGGGCTATACT	32,861–32,880
HAdV-3-Fiber-1F	CTTCCTACCAGCAGCACCTC	31,228–31,247
HAdV-3-Fiber-1R	CGTGGGGAGAGATTGGTGTA	32,419–32,438
HAdV-4-Fiber-1F	CTTCCCAGCTCTGGTACTGC	31,349–31,368
HAdV-4-Fiber-1R	GGAGGGTGGAGGGAAAATAA	32,849–32,868
HAdV-7-Fiber-1F	GAAATTTTCTCCCAGCAGCA	31,100–31,119
HAdV-7-Fiber-1R	ATTGGCTCGCTTTGAAACTG	32,392–32,411
HAdV-55-Fiber-1F	GAAATTTTCTCCCAGCAGCA	30,626–30,645
HAdV-55-Fiber-1R	AGATTGGCTCGCTCTGAAAC	31,921–31,940

^*a*^The nucleotide positions indicated are those according to HAdV-2, 3, 4, 7, 55 (GenBank accession numbers AC_000007, AY599834, KF006344, JX625134, FJ643676).

### Sequence analyses and phylogenetic calculations

Sequencher software (Version 5.0; Gene Codes Corporation, USA) was used to edit and assemble the raw sequence data. The BLASTn program (National Center for Biotechnology Information, Bethesda, MD, USA) was used to identify the homologous nucleotide sequences in the GenBank database. Phylogenetic trees were generated by using the neighbour-joining method and the Kimura-2-parameters model implemented in the MEGA program (Version 5.03) [16]. The reliability of the tree at each branch node was estimated by 1000 bootstrap replicates. Bootstrap values greater than 80% were considered statistically significant for grouping.

## Results

### Type identification of HAdVs

**Fig 1:**
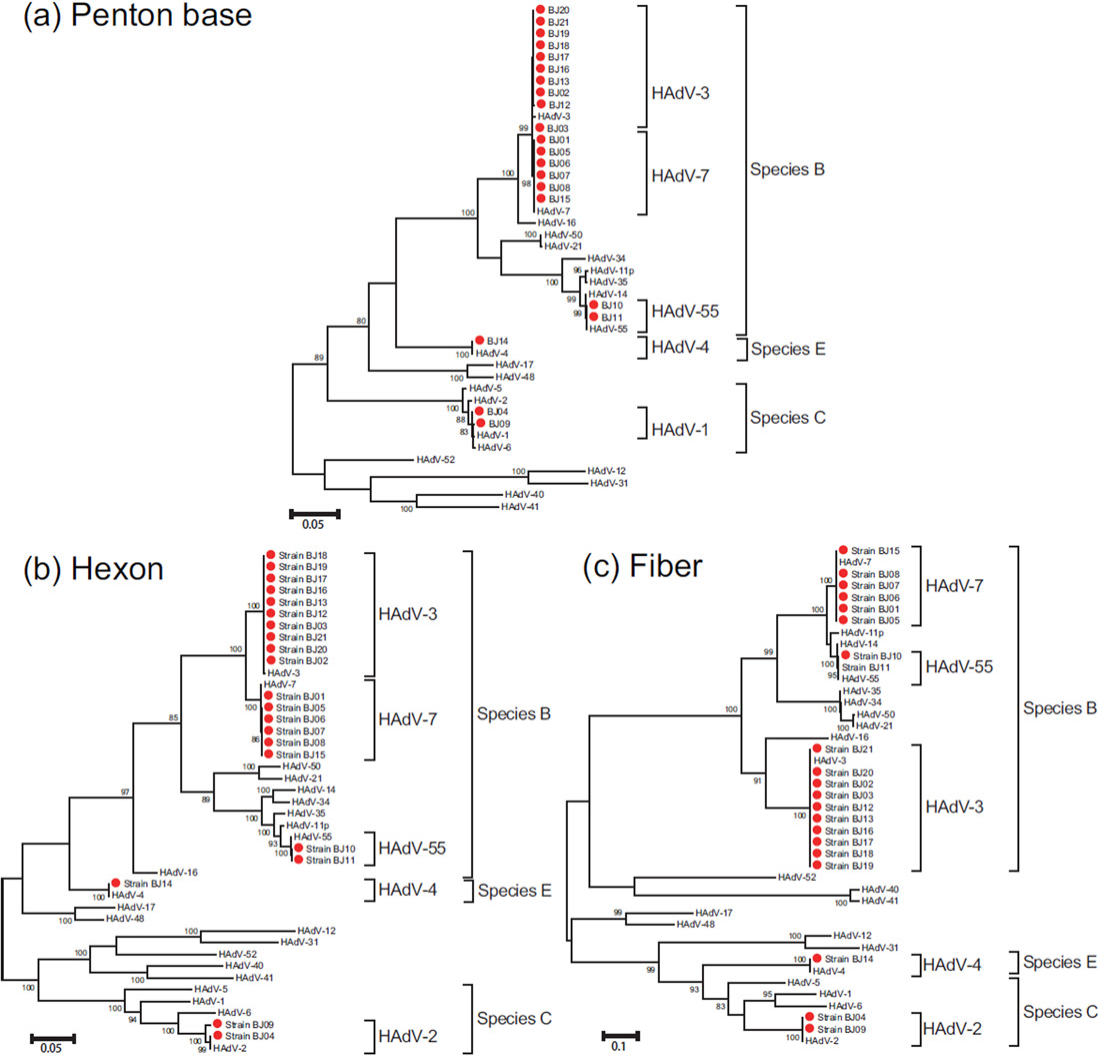
Phylogenetic analysis of Beijing adenovirus isolates based on the entire penton base, hexon, and fiber gene. The phylogenetic tree was generated by using the maximum likelihood method. The red dots indicate the strains collected in Beijing in 2011–2013. The GenBank accession numbers for each HAdV are as follows: HAdV-1, AF534906; HAdV-2, AC_000007; HAdV-3, DQ099432; HAdV-4, KF006344; HAdV-5, AC_000008; HAdV-6, FJ349096; HAdV-7, JX423383; HAdV-11p, AY163756; HAdV-12, X73487; HAdV-14, AY803294; HAdV-16, JN860680; HAdV-17, HQ910407; HAdV-21, KF528688; HAdV-31, AM749299; HAdV-34, AY737797; HAdV-35, AC_000019; HAdV-40, L19443; HAdV-41, DQ315364; HAdV-48, EF153473; HAdV-50, AY737798; HAdV-52, DQ923122; HAdV-55, FJ643676.

During 2011–2013, 4617 cases of respiratory infections were identified by the surveillance project; 45 were identified as adenovirus-positive. After virus isolation, 21 adenovirus isolates were obtained from sporadic and outbreak cases of respiratory infection: 2 cases from 2011, 2 cases from 2012, and 17 cases from 2013 (Table 2). Penton base, hexon, and fiber genes from virus isolates were successfully amplified. By the phylogenetic analysis of 21 Beijing HAdV strains and 22 HAdV type strains representing 7 species, three species (species HAdV-B, HAdV-C, and HAdV-E) were identified, including HAdV-3 (species HAdV-B; 10 strains: 1 in 2011, 1 in 2012, and 8 in 2013), HAdV-4 (species HAdV-E; 1 strain in 2013), HAdV-7 (species HAdV-B; 6 strains: 1 in 2011 and 5 in 2013), HAdV-55 (species HAdV-B; 2 strains in 2013), and a undefined HAdV type with HAdV-1 (accession number: AF534906) like penton base gene and HAdV-2 (accession number: AC_000007) like hexon gene and fiber gene (species HAdV-C; 2 strains: 1 in 2012 and 1 in 2013) were identified (Fig. 1).

**Table 2:** Twenty-one cases associated with HAdV infections in Beijing during 2011–2013.

ID	Sex	Age	Case type	Clinical symptoms	Body temperature (centigrade)	Year of sample collection	HAdV types	GenBank accession number of penton base/hexon/fiber gene*
BJ01	Female	38	Inpatient	Pneumonia	38.8	2011	HAdV-7[P7H7F7]	KP270906/KM458622/KP270915
BJ02	Male	2	Inpatient	Upper respiratory tract infection	38.5	2011	HAdV-3[P3H3F3]	KP270907/KM458623/KP270916
BJ03	Male	8	Outpatient	Upper respiratory tract infection	38.5	2012	HAdV-3[P3H3F3]	KP270908/KM458624/KP270917
BJ04	Male	1	Outpatient	Bronchitis	38.7	2012	Undefined[P1H2F2]	KP270909/KM458625/KP270918
BJ05	Male	18	Inpatient	Pneumonia	40.0	2013	HAdV-7[P7H7F7]	
BJ06	Male	18	Inpatient	Pneumonia	39.0	2013	HAdV-7[P7H7F7]	
BJ07	Female	24	Outpatient	Upper respiratory tract infection	38.0	2013	HAdV-7[P7H7F7]	KP270910/KM458626/KP270919
BJ08	Male	17	Inpatient	Pneumonia	39.0	2013	HAdV-7[P7H7F7]	
BJ09	Female	1	Outpatient	Upper respiratory tract infection	38.5	2013	Undefined[P1H2F2]	KP270911/KM458627/KP270920
BJ10	Male	26	Inpatient	Pneumonia	39.5	2013	HAdV-55[P14H11F14]	KP270912/KM458628/KP270921
BJ11	Male	37	Inpatient	Pneumonia	38.2	2013	HAdV-55[P14H11F14]	
BJ12	Female	34	Inpatient	Pneumonia	38.4	2013	HAdV-3[P3H3F3]	
BJ13	Male	20	Outpatient	Upper respiratory tract infection	39.5	2013	HAdV-3[P3H3F3]	
BJ14	Male	6	Outpatient	Upper respiratory tract infection	38.7	2013	HAdV-4[P4H4F4]	KP270913/KM458629/KP270922
BJ15	Female	6	Outpatient	Upper respiratory tract infection	39.5	2013	HAdV-7[P7H7F7]	
BJ16	Male	12	Outpatient	Upper respiratory tract infection	Unknown	2013	HAdV-3[P3H3F3]	
BJ17	Male	12	Outpatient	Upper respiratory tract infection	Unknown	2013	HAdV-3[P3H3F3]	
BJ18	Male	12	Outpatient	Upper respiratory tract infection	Unknown	2013	HAdV-3[P3H3F3]	
BJ19	Female	12	Outpatient	Upper respiratory tract infection	39.0	2013	HAdV-3[P3H3F3]	KP270914/KM458630/KP270923
BJ20	Female	12	Outpatient	Upper respiratory tract infection	38.5	2013	HAdV-3[P3H3F3]	
BJ21	Male	12	Outpatient	Upper respiratory tract infection	40.0	2013	HAdV-3[P3H3F3]	

*Note: Entire penton base, hexon, and fiber gene sequences obtained.

Based on the nucleotide alignments of their penton base, hexon, and fiber gene sequences, the HAdV-3, HAdV-4, HAdV-7, and HAdV-55 strains, isolated in Beijing during 2011–2013 were more conserved, with nearly 100% nucleotide identity to the corresponding type of HAdV. In penton base, hexon, and fiber genes, the identities between HAdV-7 strains from 2011 and 2013 were about 99.9%-100% with 0–3 nt substitutions, and HAdV-3 strains from 2011 to 2013 also had the highest sequence similarity (99.8–100%, 0–3 nt substitutions). While strain BJ04 (isolated in 2012) and BJ09 (isolated in 2013) that belong to the undefined HAdV type showed high identities in penton base gene (99.7%, 5 nt substitutions) and fiber gene (99.9%, 2 nt substitutions), but shared only 98.9% nucleotide sequence identity with 33 nt substitutions in hexon gene, indicating slight variation. According to the chronological distribution of the viruses, 9 representative HAdV strains [HAdV-3 (3), HAdV-4 (1), HAdV-7 (2), HAdV-55 (1), and the undefined HAdV type (2)] were selected to deposit in the GenBank nucleotide sequence database (penton base: KP270906- KP270914; hexon: KM458622- KM458630; fiber: KP270915- KP270923), and also for further analysis (Table 2).

### Clinical profile of HAdV infected patients

All the 21 patients who got HAdVs infections presented fever in between 38.2 to 40.0 degrees, seven (33.3%) of 21 patients had radiographic evidence of pneumonia, one patient (4.8%) had bronchitis, and others 13 patients (61.9%) had only upper respiratory tract infection symptoms such as cough and runny nose (Table 2). Among them, HAdV-55 infections (2 cases) and HAdV-7 infections (4 of 6 cases) seems led to patients of severe symptoms (pneumonia), while HAdV-3 and HAdV-4 infection caused minor symptoms (symptoms of upper respiratory tract infection or bronchitis), with only one HAdV-3 infection causing pneumonia. It is worth noting that the two patients infected with the undefined HAdV type appeared to have only mild symptoms such as fever and cough, and both patients affected by this recombinant virus are infants (below 1 years old), while patients infected with other HAdVs are all teenagers or adults.

### Strains BJ04 and BJ09 are recombinants between HAdV-2 and HAdV-1 representing a novel HAdV genotype

**Fig 2:**
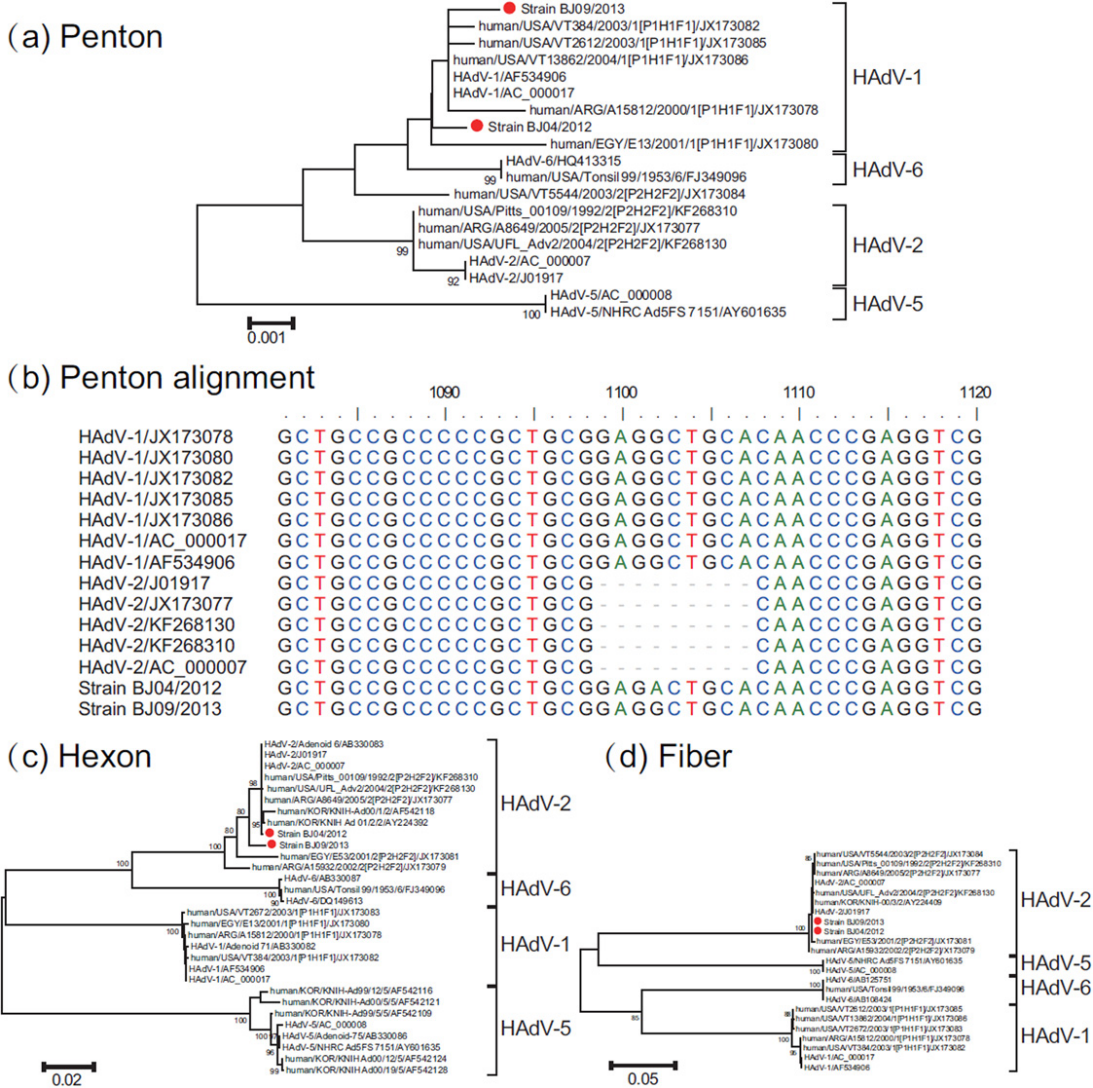
Phylogenetic analysis of species HAdV-C strains (undefined HAdV type) collected in Beijing. (a) the entire penton gene; (b) sequence alignment of partial penton gene; (c) the entire hexon gene; (d) the entire fiber gene. The phylogenetic trees were generated by using the maximum-likelihood method with 1000 replicates. Beijing strains are indicated by red dots.

In order to further identify possible recombination events, phylogenetic analyses based on the penton base, hexon, and fiber gene sequences were used to analyse the relationship of BJ04 and BJ09 strains in this study as well as with HAdV-C strains in the GenBank database. (Fig. 2).

In the penton base sequences, strain BJ04 and BJ09 were close to the corresponding sequences of the HAdV-1 strains in the GenBank database, and exhibited less similarity with the HAdV-2 prototype strain recorded in the GenBank database (Fig. 2a). And from the nucleotide sequence alignment based on the penton base gene (Fig. 2b), strains BJ04 and BJ09 are a little more like HAdV-1 sequences, due to HAdV-2 sequences having a 9-nt deletion from nt 1099 to nt 1107. In the hexon gene and fiber gene, the sequences of strain BJ04 and strain BJ09 clustered with that of HAdV-2 (Fig. 2c and 2d). These findings indicated that strain BJ04 and BJ09 may be recombinants having penton base gene of HAdV-1, and hexon gene and fiber gene of HAdV-2. In addition, recombination was not found among any of the other strains of HAdV-1 and HAdV-2 available in the GenBank database.

### Phylogenetic analysis of other HAdV-3, HAdV-4, HAdV-7, and HAdV-55 Beijing strains showed relative genome stability

**Fig 3:**
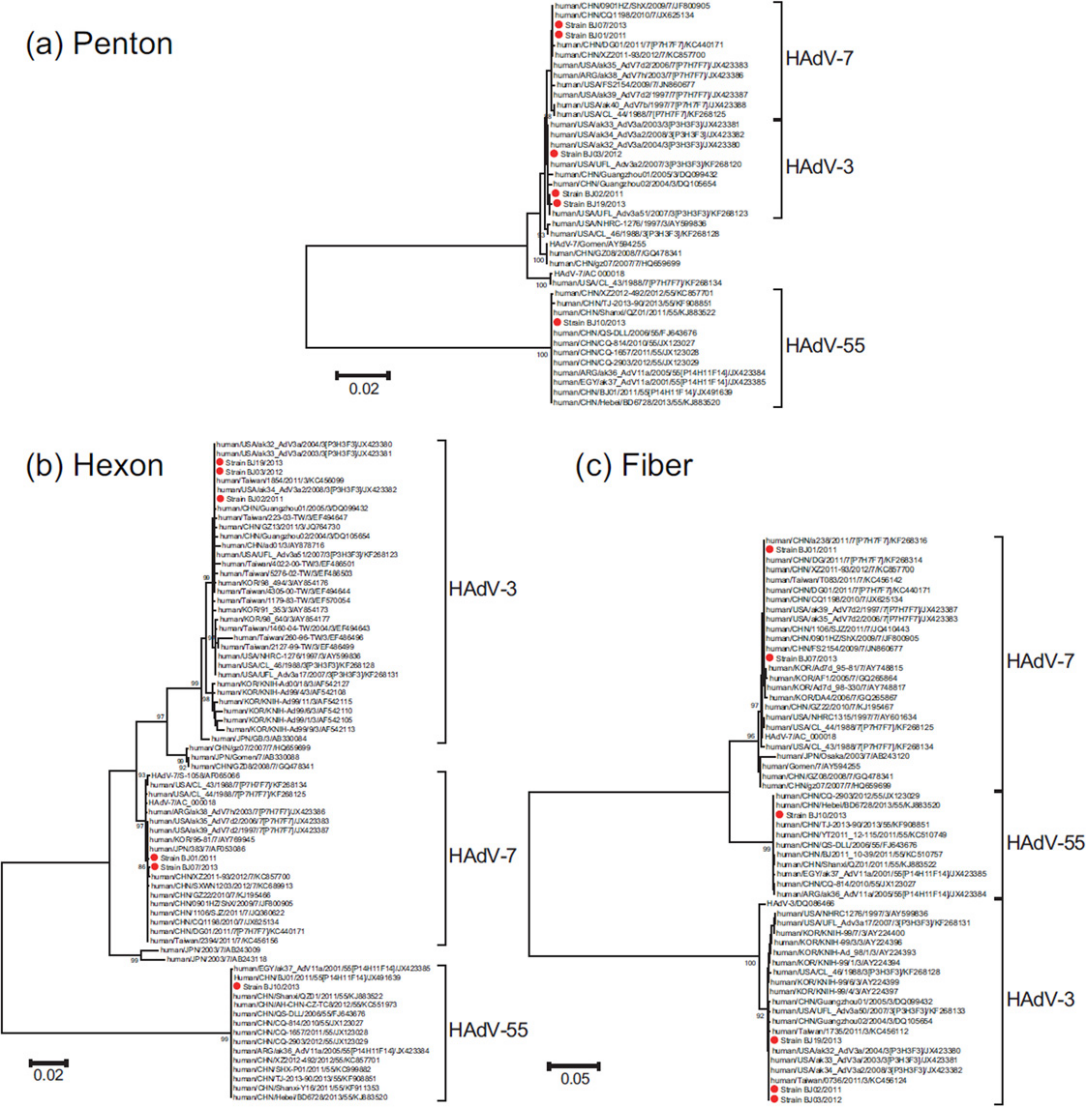
Phylogenetic analysis of species HAdV-B strains (HAdV-3, 7, 55) collected in Beijing. (**a**) the entire penton gene; (**b**) the entire hexon gene; (**c**) the entire fiber gene. The phylogenetic trees were generated by using the maximum-likelihood method with 1000 replicates. Beijing HAdV-3, 7, and 55 strains are indicated by red dots.

**Fig 4:**
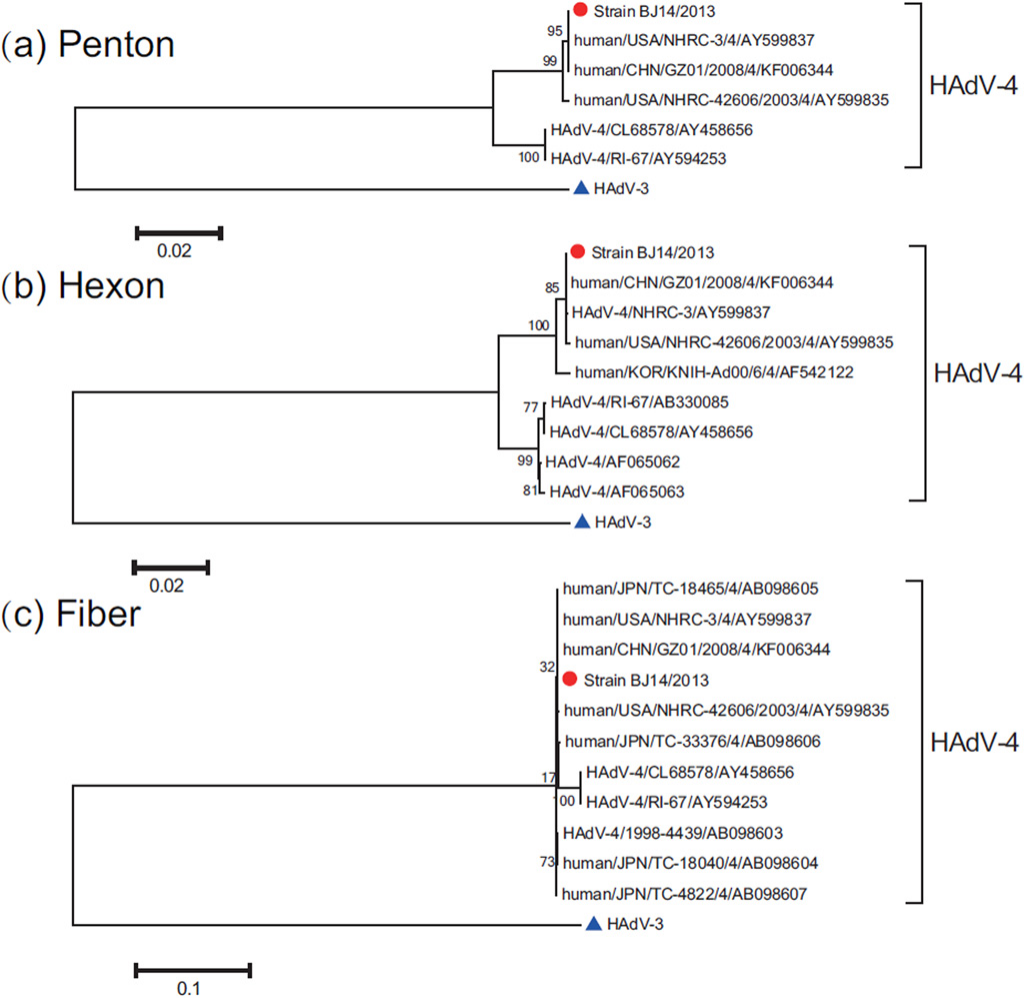
Phylogenetic analysis of species HAdV-E strains (HAdV-4) collected in Beijing. (a) the entire penton gene; (b) the entire hexon gene; (c) the entire fiber gene. The phylogenetic trees were generated by using the maximum-likelihood method with 1000 replicates. Beijing HAdV-4 strains are indicated by red dots. The HAdV-3 strain (GenBank accession number DQ099432) with blue triangle was used as an outgroup.

The penton base, hexon, and fiber gene sequences of 7 representative Beijing HAdV strains in the present study belonging to HAdV-B (including HAdV-3, HAdV-7, and HAdV-55) and HAdV-E (including HAdV-4) were aligned and analyzed with sequences identified in the mainland of China [HAdV-3 (from 2004 to 2011), HAdV-4 (from 2008), HAdV-7 (from 2009 to 2012), and HAdV-55 (from 2006 to 2013)] and sequences from other countries and regions [HAdV-3 (from 1988 to 2011), HAdV-4 (from 2003), HAdV-7 (from 1988 to 2011), and HAdV-55 (from 2001 to 2005)] from Taiwan, Argentina, Egypt, Korea, Japan, and the USA (Figs. 3, 4). Beijing HAdV strains (representing 4 types) clustered with the sequences from the different provinces of the mainland of China and other HAdVs within each corresponding type, and exhibited the highest similarity between those sequences. This indicated that these 4 HAdV types’ genomes (HAdV-3, HAdV-4, HAdV-7, and HAdV-55) are stable.

## Discussion

This study documents the HAdV types associated with respiratory infection in Beijing during 2011–2013 through the surveillance project, and viruses in species HAdV-B (HAdV-3, 7, 55), HAdV-C (undefined HAdV type), and HAdV-E (HAdV-4) were identified. These results are similar to those of a previous study performed between 2005 and 2010 in Beijing [17], and confirmed that at least 5 HAdV types were co-circulating and were the causative agents of respiratory tract infections in recent years in Beijing. In addition to Beijing, these HAdVs were also most frequently detected from acute respiratory tract disease cases in other cities of China such as Guangzhou in Central South China [18] and Lanzhou City in Northeast China [19], which indicated that these HAdV types are widely distributed in China. This study is also consistent with reports from Argentina [20], USA [21], Egypt [22], and Korea [23].

In this study, a multiplex one-step RT-PCR was performed to screen for 15 different respiratory viruses using a commercial kit (Seeplex RV 15 ACE Detection kit), and HAdV is one of the target viruses. The reason for the positivity rate of multiplex one-step RT-PCR (45 adenovirus positive) being higher than that of the viral isolation (21 positive), may be mainly due that this method is more sensitive than viral isolation method for HAdVs, and an other possible reason may be that the specimen collection is not timely, etc.

The comparison results of the penton base, hexon, and fiber gene sequences between the Beijing HAdV-3, HAdV-4, HAdV-7, and HAdV-55 strains in this study and the strains from the GenBank database indicated significant conservation and stability of the sequences within the genome across time and space. This genome stability of HAdV-3, 4, and 7 was also reported in earlier studies, showing that HAdV-3 has displayed a relatively stable genome for more than 50 years [24]; the genomes of the HAdV-4 and HAdV-7 strains are also remarkably conserved, albeit only extending for at least 20 years [25]. This characteristic has also been found for other HAdVs such as HAdV-5, whose genome was found to be stable even beyond the 45 years of its circulation in the population [26]. Limited mutations and infrequent recombination may contribute to the long-term success of HAdV-3, 4, and 7 vaccines.

HAdV-55 infection has gained attention in the last decade. Genomics and bioinformatics data indicate that HAdV-55 (earlier name HAdV-11a) is an emergent respiratory pathogen due to recombination between HAdV-11 and HAdV-14 [13,14]. As a newly identified acute respiratory disease pathogen, HAdV-55-associated outbreaks were reported to have occurred in military camps of Singapore and Turkey in 2005[27], and in a senior high school in the Shaanxi province of China in 2006 [13]. Since 2008, this pathogen has been isolated from cases of respiratory infection and community-acquired pneumonia among adults in Beijing, Hebei, Shandong, Chongqing, and Gansu provinces of China [28–30]; it was also isolated among military trainees in Hebei province of China in 2012 [30]. This may raise a serious public health concern, because HAdV-55 has the potential to spread and cause severe epidemics in China, and it may become a major etiological agent causing pneumonia among the Chinese population. In this study, two patients who were affected with HAdV-55 also appeared to have relatively severe symptoms (clinical diagnosis was pneumonia), highlighting the significance of HAdV-55 as being an increasing cause of respiratory illness especially pneumonia. Therefore, efforts should be focused on a HAdV-55 vaccine because of its stable genome. In addition, the genome stability of these HAdVs is also a desired property in the application of these viruses as gene delivery vectors.

In contrast to the apparent stability of the genomes of HAdVs in general, they are also known to undergo recombination. Recombination is a well-known feature in HAdV genetics and is one of the most important factors driving the evolution of HAdVs [31]. In this study, the phylogenetic analysis indicated that strain BJ04 and strain BJ09 may have recombined between HAdV-1 genome (penton base gene) and HAdV-2 genome (hexon and fiber genes) within species HAdV-C, which are intraspecies recombination events. Intraspecies recombinations have already been identified in many emergent HAdV pathogens, which were subsequently identified as novel type HAdVs, such as HAdV-53 to 68 [13,31,32]. In this study, strains BJ04 and BJ09 were identified as intraspecies recombinants, and this recombination pattern (P1H2F2) has not yet been found in elsewhere, which indicated that it may also be a novel HAdV type. Although the two patients affected with this undefined type HAdV appeared to have only mild symptoms (clinical diagnosis was upper respiratory tract infection), the pathogenicity remains unclear and the virus has the potential to cause serious symptoms because of its infection of children below 1-year old. Further studies on the whole genomic sequence and virulence determination including a comparison of the growth kinetics and cytopathology of the recombinant virus and the two parental strains (HAdV-1 and HAdV-2) will be required to elucidate the characteristics of this novel HAdV type.
